# Editorial: Mechanisms contributing to sleep-dependent memory generalization

**DOI:** 10.3389/fnins.2022.1106577

**Published:** 2022-12-20

**Authors:** Itamar Lerner, Praveen K. Pilly, Ahmed A. Moustafa

**Affiliations:** ^1^Department of Psychology, The University of Texas at San Antonio, San Antonio, TX, United States; ^2^Proficient Autonomy Center, Intelligent Systems Laboratory, HRL Laboratories, LLC, Malibu, CA, United States; ^3^School of Psychology, Faculty of Society and Design, Bond University, Gold Coast, QLD, Australia; ^4^Department of Human Anatomy and Physiology, The Faculty of Health Sciences, University of Johannesburg, Johannesburg, South Africa

**Keywords:** sleep, memory consolidation, generalization, regularities extraction, pattern recognition, abstraction

Contemporary research of sleep in humans and animals repeatedly show its involvement in memory consolidation (Rasch and Born, 2013). While early studies in the field were mostly concerned with the way sleep strengthens existing memories and skills (e.g., Plihal and Born, 1997), it has since become clear that sleep—particularly sleep stages known collectively as non-Rapid-Eye-Movement (non-REM) sleep—play an important role in the generalization of experience to new circumstances, including gist learning, extraction of regularities, and the development of insight into hidden patterns. The extent of these effects has nevertheless become the issue of extensive debates in the field, with questions raised regarding the conditions that allow sleep-dependent generalization to emerge, the neural mechanisms supporting it, and even how robust the effects really are (e.g., Cordi and Rasch, 2021). The current Research Topic presents studies that directly tackle some of these core questions.

Gibson et al. investigated whether theta oscillations during encoding, thought to be involved in tagging memories for subsequent sleep-dependent consolidation (Heib et al., 2015), also contribute to memory generalization. They employed the Deese-Roediger-McDermott (DRM) paradigm, where participants are exposed to a list of words that share a common theme and are later tested on how well they remember these words (“veridical memories”) and whether they mistakenly think they also saw the unpresented theme word (“generalization”). It was found that while theta oscillations in participants who stayed awake between exposure and test predicted an increase in veridical memories but reduced generalization, participants who slept during this interval showed the opposite pattern, with effects also correlated to sleep spindle density. Therefore, the results pointed to a complex interaction of theta oscillations with memory consolidation, hinting that they might tilt the balance between memory recall and generalization toward the latter when sleep is involved.

Manassero et al. also examined the effect of sleep on recall and generalization, but in the context of explicit and implicit threat detection. Participants underwent fear conditioning followed by sleep deprivation either one or seven nights after exposure, or no deprivation at all. Participants' sensitivity to threat from the conditioned stimulus (“memory recall”) or from a new, similar stimulus (“generalization”) was then examined in two ways: implicitly, by measuring skin conductance response to the presentation of the stimuli, and explicitly, by asking participants to identify the conditioned stimulus. They found that while sleep deprivation did not modulate memory recall, it increased implicit threat generalization—as long as deprivation occurred the night immediately following exposure. This study thus demonstrated, once again, a differential influence of sleep on memory recall and generalization, but one that was qualified by sleep timing and the type of memory processes probed during testing.

The implicit and explicit effects of sleep were also studied by Koroma et al. using another generalization paradigm, associative transfer. Participants first associated images of animals or similar natural elements with their respective sounds during wake, and were then exposed during either REM or non-REM sleep to some of those sounds together with the name of the corresponding animal spoken in a foreign language. Upon awakening, participants' ability to correctly associate the foreign name with the correct image (which never appeared together before) was tested. Participants tended to pick the correct association only for images that were named during non-REM sleep. This ability was implicit only; when asked to indicate the confidence in their choices (taken to reflect explicit knowledge), participants exhibited similar confidence for all words regardless of whether they were heard during sleep or not. Moreover, changes in cortical slow waves during sleep in response to the spoken words were predictive of eventual performance on these words at the subsequent test, suggesting a neural mechanism contributing to the behavioral effects.

Approaching the question of explicit vs. implicit processes from yet another angle, Lerner and Gluck centered on a particular theory about the type of generalizations that sleep facilitates. The *Temporal Scaffolding hypothesis* (Lerner and Gluck, 2018, 2019; Lerner et al., 2019) suggests that sleep is especially beneficial in facilitating explicit generalization of memories with temporal patterns. To test the hypothesis, they examined whether overnight sleep, compared to a similar wake time, would preferentially help participants identify sequential regularities that are disparate in time compared to those that are more temporally condensed. They found that sleep uniformly facilitated detection of regularities regardless of how temporally spread they were; but, at the same time, recognition of the most disparate regularities critically relied on sleep and required explicit processes (i.e., participants consciously recognizing the pattern) whereas recognition of more temporally condensed regularities could also happen during wake, and sleep only facilitated it implicitly. The results were thus generally consistent with the hypothesized predictions.

Finally, using two complementary tasks that evaluated the ability to learn category-location associations either implicitly or explicitly, Talamini et al. have shown that sleep did not contribute more than wake to generalization of hippocampus-dependent memories. Rather, sleep merely helped to strengthen the original memories, and, in some cases, a tradeoff between memory and generalization facilitation was found—albeit not one that depended on sleep. Thus, this study demonstrated the limits of sleep-dependent generalization. Note, however, that no temporal regularities were embedded in the stimuli; therefore, at least from the perspective of the temporal scaffolding hypothesis (Lerner and Gluck, 2019), a lack of explicit generalization effects was expected.

Taken together, the collection of studies revealed several common threads. First, generalization effects were highly task-dependent (cf., Lerner and Gluck, 2019); second, sleep seemed to preferentially facilitate implicit memory generalization whenever regularities embedded in the task had little to no temporal components (the only time sleep-related explicit generalization emerged was when temporal patterns were emphasized); and third, memory generalization and recall often exhibited a tradeoff, echoing previous findings in the literature (Gomez et al., 2006; Alger and Payne, 2016; Davidson et al., 2018; Lerner et al., 2021) and consistent with theories suggesting sleep serves to extract the “gist” of encoded memories while diminishing individual details (Stickgold and Walker, 2013). These repeating trends may guide future models of sleep-dependent generalization.

## Author contributions

IL wrote the first draft of the Editorial. PP and AM reviewed and edited the Editorial. All authors contributed to the article and approved the submitted version.
